# Right inferior phrenic artery pseudoaneurysm after a laparoscopic gastrectomy: a case report

**DOI:** 10.1186/s40792-019-0739-x

**Published:** 2019-12-02

**Authors:** Kaoruko Funakoshi, Yuji Ishibashi, Shuntaro Yoshimura, Ryoto Yamazaki, Fumihiko Hatao, Yasuhiro Morita, Kazuhiro Imamura

**Affiliations:** 1 Department of Surgery, Tokyo Metropolitan Tama Medical Center, 2–8–29 Musashidai, Fuchu-shi, Tokyo, 183–8524 Japan; 2 Department of Surgery, Tokyo Metropolitan Matsuzawa Hospital, 2-1-1 Kamikitazawa, Setagaya-ku, Tokyo, 156-0057 Japan

**Keywords:** Pseudoaneurysm, Right inferior phrenic artery, Laparoscopic gastrectomy, Gastric cancer

## Abstract

**Background:**

Ruptured pseudoaneurysms are a rare complication of gastrectomy, but when they do develop, they are often fatal. We presented herein the first report of a case of pseudoaneurysm arising from the right inferior phrenic artery (RIPA) after a laparoscopic gastrectomy.

**Case presentation:**

A 61-year-old male patient underwent a laparoscopic distal gastrectomy and D1+ lymph node dissection with Roux-en-Y reconstruction for early gastric cancer. He was discharged on postoperative day (POD) 9 without any complications, such as anastomotic or pancreatic leakage. On POD 19, he was referred to the emergency room for upper abdominal pain. Enhanced abdominal computed tomography revealed a 60 × 70 mm hematoma, indicating intra-abdominal bleeding and a 10-mm pseudoaneurysm in the RIPA. Selective digital subtraction angiography confirmed the presence of a pseudoaneurysm in the RIPA, which was embolized using multiple microcoils. Thereafter, no clinical signs were observed, and the patient was discharged from the hospital 15 days after angiography without any recurrence of bleeding. We hypothesized that the cause of the pseudoaneurysm was mechanical vascular injury due to the thermal spread of the ultrasonically activated devices (USADs) during lymphatic node dissection.

**Conclusion:**

Given the thermal spread of USADs, safe and appropriate lymph node dissection based on precise anatomical knowledge is crucial to preventing postoperative pseudoaneurysms.

## Introduction

Pseudoaneurysms result from the partial to complete disruption of the vascular wall and ultimately lead to hemorrhage contained by the adventitia of the vessel wall or the perivascular soft tissues [1]. Inferior phrenic artery (IPA) pseudoaneurysms are a very rare form of visceral pseudoaneurysm. Ruptured pseudoaneurysms are also a rare complication sometimes reported after a gastrectomy [2], but when they do develop, they are often fatal. We presented herein the first report of a case of a pseudoaneurysm arising in the right inferior phrenic artery (RIPA) after a laparoscopic gastrectomy.

## Case report

**Fig. 1:**
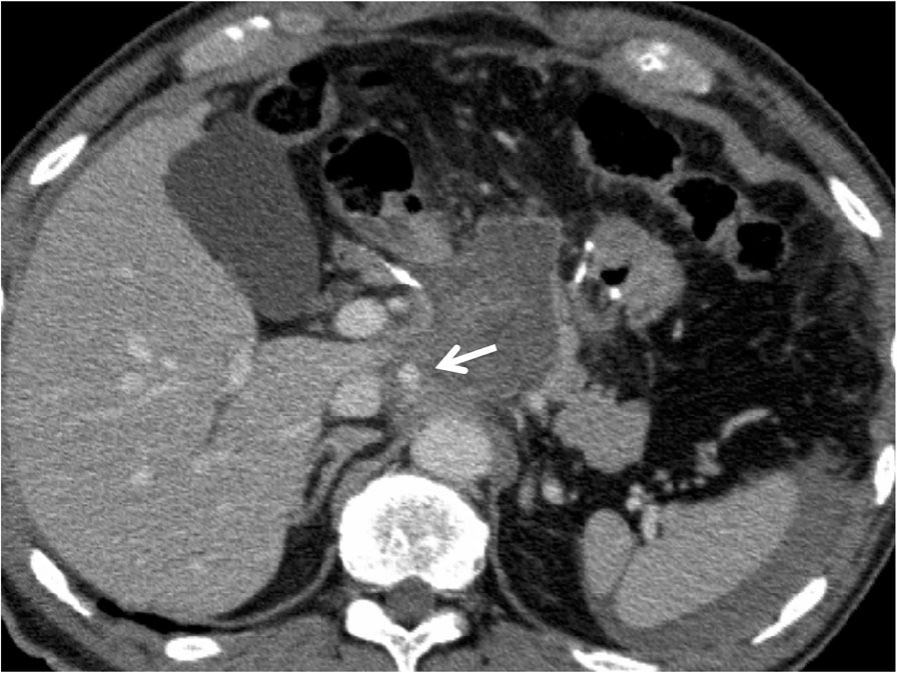
CT revealed a 60 × 70-mm hematoma and a 10-mm pseudoaneurysm (arrow) in the RIPA

**Fig. 2:**
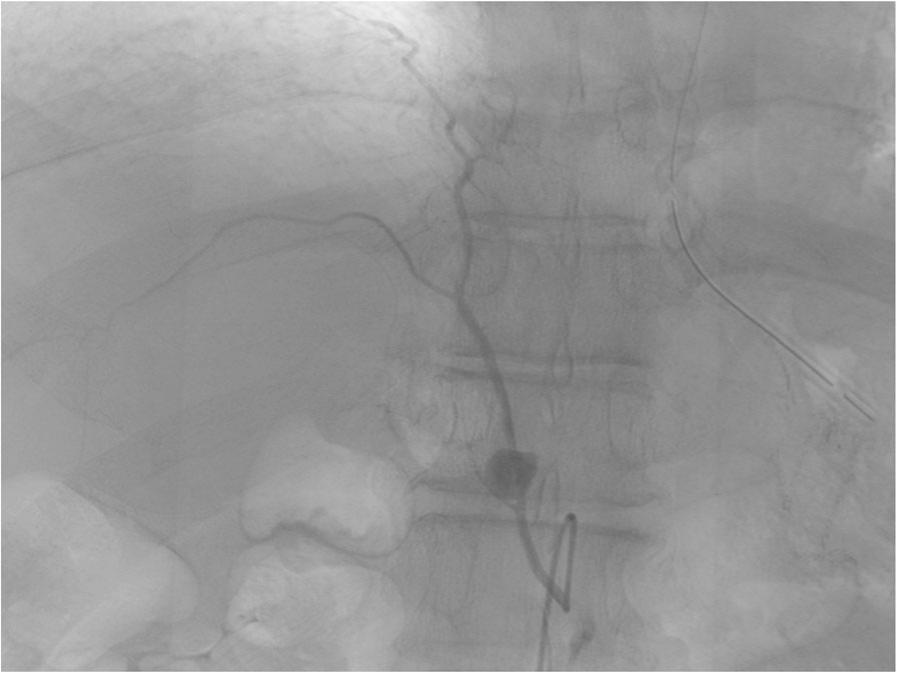
Angiography showed a pseudoaneurysm in the RIPA

A 61-year-old male patient underwent a laparoscopic distal gastrectomy and D1+ lymph node dissection with Roux-en-Y reconstruction for early gastric cancer. He was discharged on postoperative day (POD) 9 without any complications, such as anastomotic or pancreatic leakage. On POD 19, he was referred to the emergency room for upper abdominal pain. Guarding and rebound tenderness were denied. Serum biochemistry showed a white blood cell count of 16.2 × 10^3^/μL, red blood cell count of 396 × 10^4^/μL, and hemoglobin 11.2 g/dL. Enhanced abdominal CT revealed a hematoma 60 × 70 mm in diameter, indicating intra-abdominal bleeding, and a 10-mm pseudoaneurysm in the RIPA (Fig. 1). A selective digital subtraction angiography confirmed the presence of a pseudoaneurysm in the RIPA (Fig. 2), which was cannulated and successfully embolized using multiple microcoils. After embolization, there were no clinical signs, and the patient was discharged from the hospital 15 days after the angiography without any recurrence of bleeding.

## Discussion

Iatrogenic pseudoaneurysms can develop as a result of (a) mechanical vascular injury during the dissection or removal of lymph nodes and connective tissue due to a malignancy; (b) the digestion of the arterial vessels resulting from a pancreatic, biliary, or enteric fistula; or (c) local sepsis [3]. Pseudoaneurysms after abdominal surgery are a rare complication and most often occur after hepatobiliary pancreatic surgery. The development of pseudoaneurysms after gastric surgery is rare, with postoperative pseudoaneurysm hemorrhages developing in only 0.17% of patients undergoing a radical gastrectomy [2]. Pseudoaneurysms can be fatal; hence, early diagnosis and proper treatment are important to improve the prognosis. A falling hemoglobin level or a low-grade fever persisting for 2 to 3 weeks postoperatively should raise the suspicion of local sepsis with the potential for pseudoaneurysm development [4]. Recent interventional techniques using arterial embolization or stent grafts have been proposed as alternatives to surgical repair and offer real advantages in terms of survival [5].

In the present case, the RIPA pseudoaneurysm developed after a laparoscopic distal gastrectomy for gastric cancer. IPA pseudoaneurysms are very rare, with only nine cases (including our case) thus far reported (Table 1) [6–13]. Furthermore, IPA pseudoaneurysms after a gastrectomy are also extremely rare, with only two cases (including our case) reported to date. The present study is the first report of a case of ruptured RIPA pseudoaneurysm after a gastrectomy. In our case, the patient did not develop anastomotic leakage, pancreatic leakage, or intra-abdominal infection after surgery; we therefore assumed that the cause of the pseudoaneurysm was a mechanical vascular injury occurring during the dissection of the celiac artery lymph node (lymph node No.9), which is adjacent to the IPA and must be dissected via D1+ or D2 lymph node dissection in a gastrectomy for gastric cancer [14, 15].

**Table 1:** Clinical characteristics of patients with a ruptured inferior phrenic artery pseudoaneurysm

Author	Gender	Age	Site	Symptoms	Cause	Initial surgery	Treatment	Outcome
Shirai [6]	M	10	Right	Abdominal pain	Surgery	Coarctectomy	Laparotomy	Alive
Toyoda [7]	F	90	Right	Abdominal pain	Surgery	Coarctectomy	Embolization	Alive
Lee [8]	M	29	Left	Shock	Blunt trauma	No	Laparotomy	Death
Harman [9]	M	41	Unknown	Active bleeding	Surgery	Liver transplant	Embolization	Alive
Arora [10]	M	55	Left	Abdominal pain	Chronic pancreatitis	No	Embolization	Alive
Salem [11]	F	19	Right	Abdominal pain	Acute pancreatitis	No	Embolization	Alive
Namikawa [12]	M	56	Right	Low blood pressure	Surgery	Open completion gastrectomy	Embolization	Alive
Gunjan [13]	M	43	Left	Shock	Chronic pancreatitis	No	Embolization	Alive
Our case	M	61	Right	Abdominal pain	Surgery	Laparoscopic gastrectomy	Embolization	Alive

**Fig. 3:**
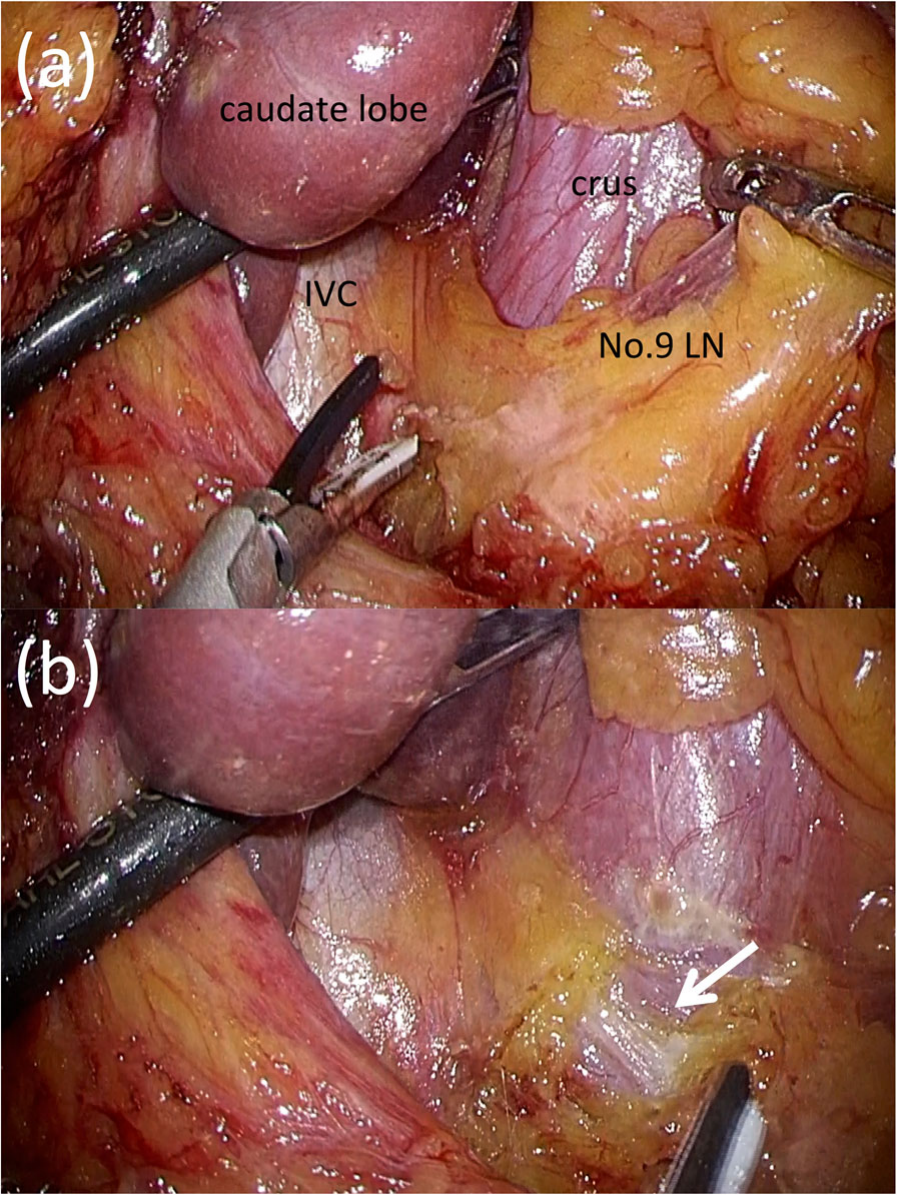
Intraoperative findings. **a** Before lymph node dissection. **b** After lymph node dissection. The RIPA was exposed (arrow)

**Fig. 4:**
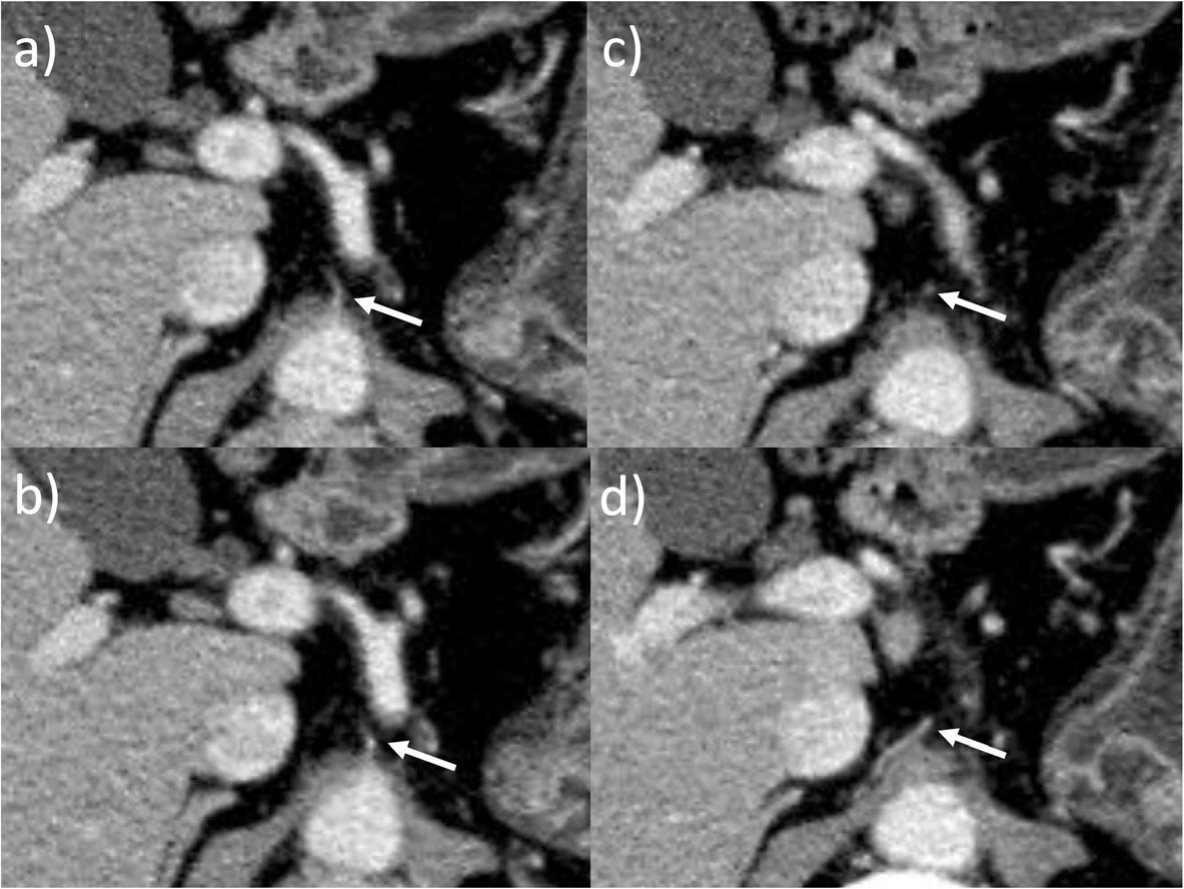
Preoperative CT. **a** The RIPA (arrow) originated from the aorta without a common trunk. **b**, **c** The RIPA ran close to the celiac artery. **d** The RIPA ran across and in front of the crus

In laparoscopic surgery, ultrasonically activated devices (USADs) are widely used for cutting, and when using USADs, precautions must be taken against lateral thermal damage to surrounding tissues that could lead to inadvertent trauma to the adjacent organs, including vessels [16]. When we reviewed the operation video, we did not observe any obvious mechanical damage to the RIPA but were unable to rule out potential damage to the RIPA due to the thermal spread of the USAD (Fig. 3). In retrospect, the following points can be adduced as possible causes of the pseudoaneurysm: (1) the flexible camera provided a very good surgical view which may have encouraged more extensive D1+ lymph node dissection than was necessary, thereby exposing the RIPA to damage; and (2) insufficient attention was given to the anatomical position of RIPA during the dissection of lymph node No.9. The RIPA is a thin vessel and difficult to recognize during lymph node dissection; therefore, the precise, preoperative localization of the RIPA is important during the dissection of lymph node No.9. The right and left IPA develop upward and laterally anterior to the crus of the diaphragm and terminate on the abdominal surface of the respective domes of the diaphragm [17]. Aslaner et al. reported that the right and left IPA were divided into two groups, those originating from a common trunk (29.5%) and those without a trunk having a different, independent origin (70.5%). In cases where IPA have a common trunk, the trunk originates from the aorta (16.4%), celiac artery (12.6%), renal artery (0.4%), or left gastric artery (0.1%). In cases where the RIPA and left IPA have disparate origins, the RIPA originates in the celiac artery (30.7%), aorta (25.2%), right renal artery (10.4%), left gastric artery (4.1%), or common hepatic artery (0.1%) [18]. In the present case, the RIPA originated from the aorta without a common trunk and ran close to the celiac artery and across and in front of the crus (Fig. 4). We surmised that in cases where the RIPA runs close to the vessels, such as the left gastric artery, common hepatic artery, or celiac artery, mechanical damage is likely to occur during lymph node dissection and lead to pseudoaneurysm development. Therefore, in these cases, closer attention should have been given to its position during dissection to avoid inflicting damage. After our experience with this case, we now routinely confirm the anatomy and direction of the RIPA on thin-section, arterial-phase dynamic CT.

## Conclusions

We reported a case of RIPA pseudoaneurysm following a laparoscopic distal gastrectomy. Given the thermal spread of USADs, safe and appropriate lymph node dissection based on precise anatomical knowledge is important to prevent postoperative pseudoaneurysms.

## Data Availability

The data are not available for public access because of patient privacy concerns but are available from the corresponding author on reasonable request.

## Abbreviations

RIPA: Right inferior phrenic artery
POD: Postoperative day
IPA: Inferior phrenic artery
USADs: Ultrasonically activated devices
